# Parameter Optimization of RB-SiC Polishing by Femtosecond Laser

**DOI:** 10.3390/ma16041582

**Published:** 2023-02-14

**Authors:** Tingkai Yang, Changhua Liu, Tao Chen, Meng Shao, Chun Jiang, Changzheng Lu, Shijun Song

**Affiliations:** 1 Changchun Institute of Optics, Fine Mechanics and Physics, Chinese Academy of Sciences, Changchun 130033, China; 2 University of Chinese Academy of Sciences, Beijing 100049, China

**Keywords:** reaction-boned silicon (RB-SiC), femtosecond laser, heat conduction, single-pulse ablation

## Abstract

Reaction-boned silicon carbide (RB-SiC) is considered a new material for large lightweight ground-based space telescopes due to its high specific stiffness, low thermal deformation, and excellent optical quality. The excellent mechanical properties of RB-SiC result in the low efficiency of traditional polishing and mechanical polishing. In this paper, a polishing method for RB-SiC based on a femtosecond laser is proposed to improve surface quality. A theoretical heat conduction model was established in the process of femtosecond laser irradiation of SiC. We analyzed the ablation type and calculated the single-pulse ablation threshold of SiC, which verified the feasibility of femtosecond laser polishing. Further, the effects of polishing parameters on the polished surface quality were analyzed by a series of experiments, and the optimal parameters were selected. It was observed to improve polishing efficiency and can replace the intermediate steps of traditional mechanical polishing.

## 1. Introduction

Due to the development of space optics, the performance and quality of optical components are significantly affected by the surface quality and texture [1]. Therefore, the machining of high-precision optical components has become the focus of research. The reaction-bonded silicon carbide is expected to become one of the most excellent and viable space optical materials due to its excellent conductivity [2]. However, because of its high hardness, the high-precision surface polishing of RB-SiC has always been a difficult processing problem [3].

Nano-scale polishing of optical components can be realized by traditional polishing methods, including abrasive polishing [4,5], wet polishing [6], magnetorheological polishing [7], and ion beam machining [8]. H. Cheng et al. put forward a magnetorheological fishing (MRF) [9] process for aspheric silicon carbide mirrors. The surface roughness decreased from 34.39 nm to 26.74 nm after 20 h of pre-polishing and then reached 1.14 nm after 50 h of fine polishing using the MR fluid with diamond power as the nonmagnetic particle. Zewei Yuan et al. proposed a polishing method for 4H-SiC wafers. In the process of mechanical polishing, the diamond abrasive of 5 μm, 2 μm and 1 μm was used to polish the surface for 30 min each to reduce the surface roughness to about 40 nm, and then the roughness was reduced to 0.35 nm by photocatalysis-assisted chemical mechanical polishing (PCMP). The material removal rate is about 0.95 μm/h [10]. Akihisa Kubota et al. used micron-sized diamond abrasive grains for high-precision mechanical polishing of diamond substrates, and the average roughness (Ra) ranging from ~0.1 nm to 0.3 nm was easily obtained by controlling the process parameters [11]. The surface roughness at the nanoscale can be obtained by traditional mechanical polishing methods, but these methods are complex, time-consuming, and require a demanding machining environment [12].

In recent years, the femtosecond laser has become an important tool for the new generation of high-precision polishing because of its excellent focusing ability and extremely high instantaneous power [13]. However, laser polishing by laser radiation is based on the remelting of a thin surface layer by laser radiation [14]. Similar to conventional polishing, laser polishing is a multi-step process. Consecutive process steps are carried out from the smoothing of milling grooves (macro polishing) up to improving the gloss level (micro polishing). In addition, crucial for the choice of whether macro or micro polishing will be used are the initial surface roughness and its spatial wavelength. The sample with an initial roughness of less than 80 µm is more suitable for microscopic polishing [15]. For micro laser polishing, pulsed laser radiation is used, which is a combination of remelting a thin surface layer smaller than 5 µm and vaporizing micro edges. In contrast to macro laser polishing, micro laser polishing is a discrete rather than a continuous remelting process. In order to better understand the mechanism of femtosecond laser processing, many scholars have conducted extensive research [14,16,17]. In recent research, A. Temmler analyzed the effect of pulse duration and repetition frequency on micro-roughness in the laser micro-polishing of AISI 410 stainless steel. When the frequency is fixed, the shorter the pulse duration, the better the polishing effect. However, the effects of pulse frequency and pulse duration on polishing results are not independent of each other. The shorter the pulse duration, the higher the repetition frequency necessary to achieve the maximum reduction of surface roughness [18]. Zhang Ru et al. established a regression model of machining groove width, depth, and material removing rate (MRR), which can accurately predict the groove width and depth formed by laser scanning the silicon carbide surface, which is of great significance for improving the roughness of the machining surface [19]. In 2006, Krzysztof et al. proposed a laser polishing method that can reduce the roughness of quartz surfaces from 1 μm to less than 1 nm [7]. Qu et al. took the lead in using a 10 ns pulsed laser to micro-polish metallic nickel surface and found that the initial surface roughness is an important factor affecting the surface polishing effect [20]. Astrid et al. proposed a method of metal polishing by femtosecond laser, analyzed the influence of different parameters on the polishing effect, and reduced the micro-roughness from 0.211 µm to 0.076 µm, which represents a reduction of 64% [17]. Huang. C et al. proposed a laser-selective polishing of RB-SiC at a fluence between its two phases, they used a femtosecond laser to selectively remove the Si on the RB-SiC surface, reducing the surface roughness from 35 nm to 11.21 ± 0.26 nm [21]. It should be noted that the RB-SiC applied in femtosecond laser selective polishing (fs-LP) in this experiment was preprocessed by chemical mechanical polishing (CMP) for about 32 h with colloidal silica slurry. However, it is still a time-consuming process. So far, there has been little research on the optimization of polishing parameters for RB-SiC polishing by femtosecond laser, which is worthy of further exploration.

In this paper, a femtosecond laser polishing method for RB-SiC is proposed. In order to choose the best laser fluence to get better surface quality, the single-pulse ablation threshold of SiC was analyzed and calculated by the two-temperature model (TTM). The influence of different polishing parameters on surface roughness, which include laser fluence, equivalent pulse number (EPN), scanning interval, and scanning model, was analyzed. In addition, by optimizing the polishing parameters, the surface quality and polishing efficiency improved obviously, which laid the foundation for the subsequent polishing with higher precision.

## 2. Experimental Details

### 2.1. Materials and Experimental Conditions

**Figure 1:**
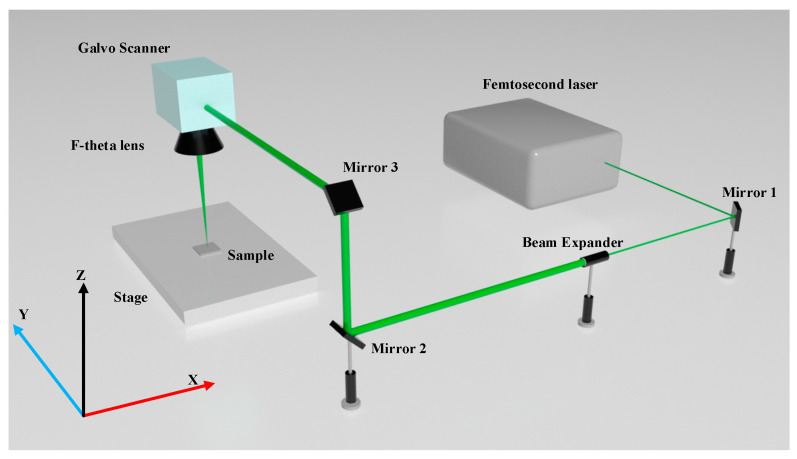
Optical path of femtosecond laser scanning.

The experiments in this work are performed on 5 mm-thick RB-SiC. The initial state of the surface after fine grinding exhibits a surface roughness of Ra = 0.2 ± 0.02 μm and a maximum roughness depth of Rz = 0.8 ± 0.08 μm. The experimental setup consists of a femtosecond laser source, Carbide from Light Conversion, which is integrated into a three-axis laser machining system. Two-dimensional processing is enabled by a galvo scanner system, the AGV10HP from Aerotech. As is shown in Figure 1, after being shaped and transmitted by the beam expander and Coude optical path (a light guide path capable of turning the laser beam to rotate with the axis system), the femtosecond laser converges to a spot on the surface of the polishing material through the F-theta mirror. Among them, mirror 2 is mounted on the x-axis of motion and can move along the x-axis direction; mirror 3 is mounted on the x- and z-axes of motion and can move along the x- and z-axes; and the oscillator and F-theta mirror are mounted on the x-, y-, and z-axes and can move along all three axes simultaneously. The optical path can achieve both a wide range of processing in the two-dimensional plane and focusing along the z-axis. In order to make the spatial layout of the experiment more reasonable, we set up mirror 1. The laser machining parameters were listed in Table 1. Furthermore, ultrasonic cleaning equipment and electrochemical cleaning equipment are prepared. Any test in this research should be completed after the first ultrasonic cleaning with deionized water, followed by anhydrous ethanol for cleaning.

The polished surface morphology was observed by a BX41M-LED industrial microscope from Olympus. The roughness of the polished surface was measured by a SJ-210 roughness meter from Mitutoyo, Japan. A sampling length of 0.8 mm is selected, and five measurements are taken along two mutually perpendicular directions. The average value is taken as the measurement result. To avoid measurement errors, measuring at the edge of the processing area should be avoided as much as possible. Due to the limited experimental conditions, we measured the samples point by point with the distance measuring lens of Keyence, selected 2601 (51 × 51) points in the measurement range of 5 × 5 mm, and simulated the surface topography based on these points.

### 2.2. Two-Temperature Model

Prior to the experiment, the TTM in the femtosecond laser ablation process was introduced, and the ablation threshold of SiC was analyzed with this model. Laser ablation mechanisms can be roughly divided into two categories, usually by the relaxation time of acoustoelectric action and laser pulses. When the laser pulse width is greater than the electroacoustic coupling time, it is called thermal equilibrium ablation, and when the laser pulse width is less than the electroacoustic coupling time, we call it unbalanced ablation [22]. During femtosecond laser processing, electrons are first accelerated and heated. Then, the lattice is heated by electron-phonon coupling, and normal energy diffuses into the materials through phonon-phonon collision [23]. This process is called non-equilibrium ablation. In this paper, the pulse duration is much less than the electroacoustic coupling time, so that unbalanced ablation occurs during the irradiation. Typically, thermal equilibrium ablation can be described by the heat conduction equation, while non-equilibrium ablation can be described by TTM. Two nonlinear differential equations of TTM can be shown as [24]:

(1) $$C_{e}\frac{\partial }{\partial t}T_{e}=K_{e}\left(\frac{\partial^{2}T_{e}}{\partial x^{2}}\right)-g\cdot \left(T_{e}-T_{l}\right)+S\left(x,t\right)$$

(2) $$C_{l}\frac{\partial }{\partial t}T_{l}=K_{l}\left(\frac{\partial^{2}T_{l}}{\partial x^{2}}\right)+g\cdot \left(T_{e}-T_{l}\right)$$

TTM describes the heat transfer among photos, electrons, and lattices using two coupled nonlinear differential equations. Here, e and l represent the electronic system and lattice system parameters, respectively. $T_{e}$ is the electronic temperature, $T_{l}$ is the lattice’s temperature; $C_{e}$ is the electronic heat capacity, $C_{l}$ is the lattice’s heat capacity; $K_{e}$ is the electronic thermal conductivity, $K_{l}$ is the lattice’s thermal conductivity, g is the characteristic parameter of the coupling between the electron and the lattice. S(x,t) is the heat source term corresponding to the laser pulse and expresses the absorption rate of the electronic system. Femtosecond laser ablation can be divided into single-pulse ablation and multi-pulse ablation. When the time interval between two pulses is much longer than the duration of one pulse, the effect between the two pulses is negligible. The repetition frequency used in this experiment is 100~500 kHz, according to which the minimum pulse interval is calculated as 2 × 10^−6^ s, much longer than 282 fs. Therefore, the polishing process in this experiment can be regarded as single-pulse ablation. The thermal absorption rate of the electronic system for single-pulse ablation can be expressed as [25]:

(3) $$S\left(x,t\right)=\sqrt{\frac{4\cdot \ln2}{\pi }}\cdot \frac{J}{t_{P}}\cdot \frac{\left(1-R\right)}{L_{p}}\cdot \exp\left(-4\cdot \ln2\cdot {\left(\frac{t-2t_{p}}{t_{P}}\right)}^{2}\right)\cdot \exp\left(-\frac{x}{L_{P}}\right)$$

In the above equations, S(x,t) describes the spatial distribution and temporal evolution of the absorbed laser energy. Here, is the laser fluence; $t_{P}$ is the full width at half maximum (FWHM) pulse duration, which is 282 fs; R is the reflectivity of SiC; $L_{P}$ is the depth of ballistic transportation; x is the depth from the surface. The laser fluence can be expressed as:

(4) $$J=\frac{2\cdot E}{\pi \cdot \omega_{0}{}^{2}}$$

Here, the beam radius $\omega_{0}$ is 10 μm, E is the pulse energy.

### 2.3. Scanning Strategy

The experimental parameters, such as laser parameters, polishing mode, and scanning parameters, will affect the polishing effect. Therefore, the parameters affecting the experimental results should be analyzed before the experiment. There are fixed ablation thresholds for different materials; even the surface morphology of the material may affect the magnitude of the ablation threshold [26]. The energy distribution of the beam used in the experiment is a gaussian distribution, which can be expressed as:

(5) $$\phi \left(r\right)=\phi_{0}e^{-2r^{2}/\omega_{0}^{2}}$$

$\phi_{0}$ is the fluence of laser beam, r is the distance from the center of beam, ϕ(r) is the laser fluence at the distance r from beam center. The relationship between ablation threshold $\phi_{th}$ and laser fluence $\phi_{0}$ can be deduced from Formula (5) as:

(6) $$D^{2}=2\omega_{0}^{2}\ln\frac{\phi_{0}}{\phi_{th}}$$

Here, D is the ablation diameter. According to Formula (6), the ablative diameter is positively correlated with the laser fluence. EPN and laser fluence jointly determine the amount and efficiency of material removal and then affect the surface quality of the sample. Equivalent pulse numbers refer to the number of laser pulses irradiated at a certain point on the sample in the scanning process, which can be expressed as:

(7) $$N=\frac{\omega_{0}}{v}\cdot f$$

**Figure 2:**
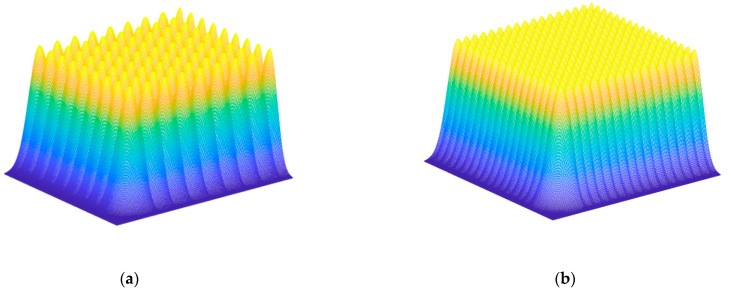
Simulation of surface topography after scanning with different pulse density: (**a**) Low pulse density (**b**) High pulse density.

**Figure 3:**
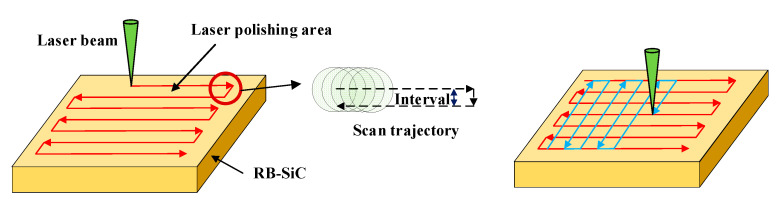
Schematic diagram of path planning for femtosecond laser polishing.

Here, v is the scanning velocity. According to Formula (6), when $\phi_{0}=e^{1/2}\phi_{th}$, the groove width is equal to the laser beam diameter. When the scanning interval is less than or equal to the groove width, full field polishing can be achieved. However, because the energy distribution of the laser beam is Gaussian, the groove scanned by the laser is V-shaped rather than U-shaped. As Figure 2a,b shows the surface topography after scanning with two different pulse densities, the higher the pulse density, the smaller the surface undulation, and the smaller the surface roughness, the better the surface quality obtained. The pulse density is related to parameters such as scanning interval, velocity, and repetition frequency. We need to adjust these parameters to obtain the best surface quality while ensuring scanning efficiency. In addition, the surface quality is also affected by scanning mode. As shown in Figure 3, we set the scanning mode to a single direction and two vertical directions to explore the impact of scanning mode on surface quality.

In addition to some experimental parameters described above that may affect the polishing effect, underwater femtosecond laser processing [27], inert gas atmosphere femtosecond laser processing [28], water-jet-guided laser processing [29], and other processing methods have also been studied in depth. Different processing environments will also have an impact on surface quality. In this paper, in order to reduce the requirements of the processing environment, experiments in the above environment are not conducted.

In the introduction, we mentioned that both pulse duration and repetition frequency have an effect on the polishing effect, and this effect is different for different materials [18]. In this experiment, the limitations of the experimental equipment do not allow us to analyze their effects, so we set these two parameters to constant values.

## 3. Results

### 3.1. Ablation Threshold of Silicon Carbide

According to TTM in Section 2.2, prior to the experiment, the ablation threshold of SiC was calculated to ensure that the femtosecond laser could be used for polishing. RB-SiC is a kind of highly dense material obtained by pressing fine particles of SiC and C bonding agent in a certain ratio into a billet and fully reacting with liquid silicon or vapor-phase silicon at high temperatures. After the reaction, the carbon inside the material is fully consumed, but there is still a small amount of silicon residue. By consulting the literature [30], it is found that the ablation threshold of silicon is much smaller than that of SiC. The laser fluence required for polishing should be greater than the silicon carbide ablation threshold. Therefore, the ablation threshold of silicon is not calculated in this paper.

Under conditions that do not affect the calculation, $C_{e},K_{e},C_{l},K_{l}$ and R were taken as constants, and they did not change with temperature [24]. Values of the thermophysical parameters of SiC are listed in Table 2.

We set the initial temperature of the material lattice and electrons not irradiated by the femtosecond laser to room temperature, which can be expressed as $T_{e}\left(x,0\right)=T_{l}\left(x,0\right)$. Since S(x,t) is the electron absorption at the surface of the material, while the electron absorption at the bottom surface of the material is 0. Therefore, the boundary conditions can be expressed as:

(8) $$-K_{e}\frac{\partial T_{e}}{\partial x}|{}_{x=0}=S\left(x,t\right)$$

(9) $$-K_{e}\frac{\partial T_{e}}{\partial x}|{}_{x=d}=0$$

**Figure 4:**
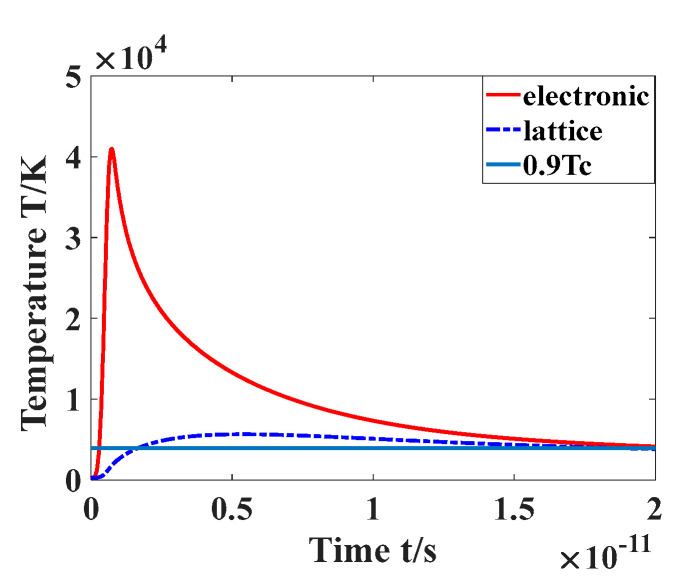
Lattice and electron temperature changes with time under threshold fluence.

Here, d is the thickness of the sample. According to the phase explosion theory [32], when the equilibrium temperature of the lattice and electrons exceeds 0.9$T_{t}$, the sample undergoes a phase explosion, and the laser fluence that produces this lattice temperature is called the ablation threshold of the material. Figure 4 shows the temperature change of the lattice and electrons when the laser fluence is equal to the ablation threshold. As is shown in Figure 4, the electron temperature rises rapidly within 100 fs and gradually transfers heat to the lattice to reach temperature equilibrium, where they eventually balance at 0.9$T_{t}$. This process takes about 20 ps. For a femtosecond laser with a frequency of 100 KHz, the interval between the two pulses is 10^−5^ s, and the action process of the first pulse is finished before the next pulse arrives, so the ablation of the two pulses can be considered independent of each other. Therefore, the ablation in this experiment can be regarded as single-pulse ablation, which validates the description of the type of ablation in Section 2.2.

**Figure 5:**
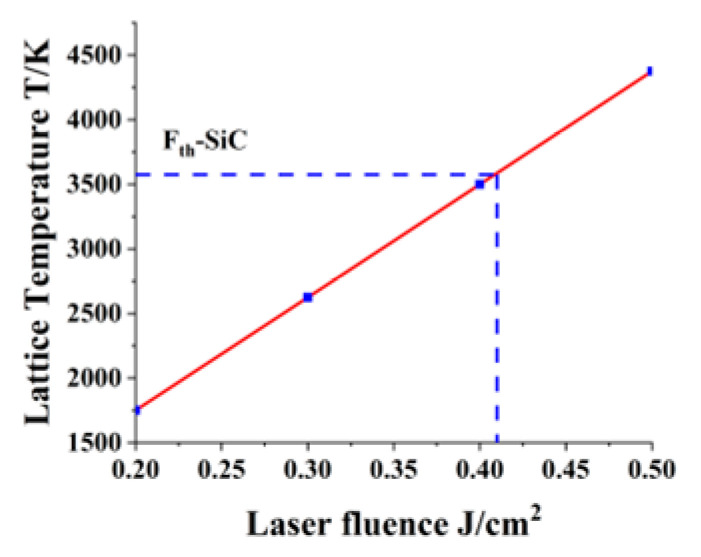
Relationship between lattice’s maximum temperature and laser fluence.

Figure 5 shows the relationship between the laser fluence and the maximum temperature of the lattice on the surface of the sample. They are linearly related, and the single-pulse ablation threshold for silicon carbide is calculated at 0.41 J/cm^2^. Yang Dong et al. [33] performed a silicon carbide ablation experiment with a femtosecond laser, whose wavelength is 800 nm and FWHM is 120 fs. They measured the ablation threshold of silicon carbide at 0.52 J/cm^2^. In this paper, some parameters have been simplified, which causes a certain gap between the theoretical calculation results and the experimental data.

**Figure 6:**
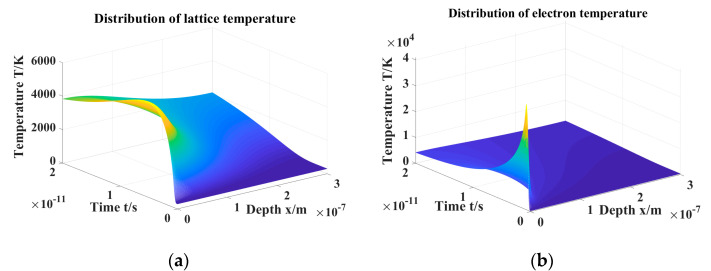
Distribution of temperature with the change of time and depth from the sample’s surface (**a**) Lattice (**b**) Electronic.

Respectively, Figure 6a,b shows the distribution of electron and lattice temperatures as a function of time and depth. By comparing the two figures, we observe that the temperature of the electron increases rapidly after laser irradiation due to its small heat capacity. At the same time, the temperature of electrons decreases rapidly with changes in depth and time due to their small thermal conductivity. In contrast, the heat capacity of the lattice is much higher than that of the electron, so its temperature rises slowly, and its maximum temperature is much lower than that of the electron. Meanwhile, because its thermal conductivity is higher, the heat around the irradiated point is greatly affected by the laser.

### 3.2. Influence of Laser Fluence and Scanning Pitch on Polishing

**Figure 7:**
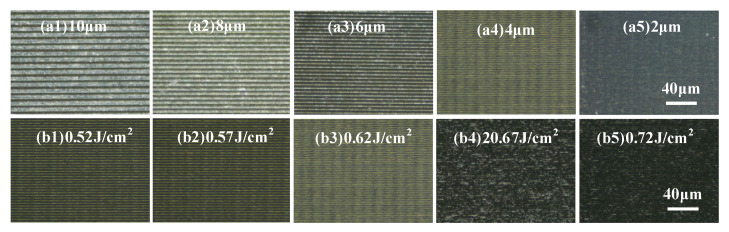
Surface morphology under different polishing parameters: (**a1**–**a5**) Different scanning intervals (**b1**–**b5**) Different laser fluence.

**Figure 8:**
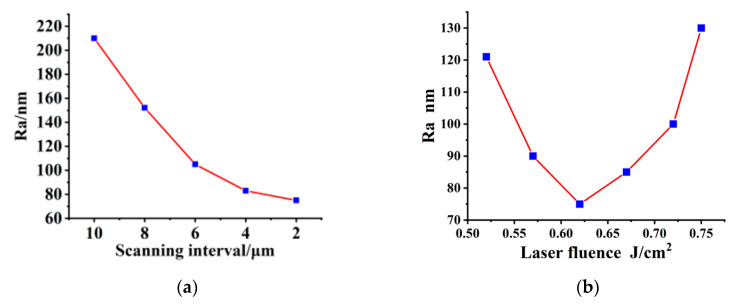
Curve of surface roughness with the change of polishing parameters: (**a**) Scanning interval; (**b**) Laser fluence.

In order to analyze the effects of scanning interval and laser fluence on surface roughness separately, we set up a series of single-variable experiments. The surface is irradiated with a scanning velocity of 150 mm/s, a repetition frequency of 100 kHz, and a laser fluence of 0.62 J/cm^2^, with different scanning intervals of 2 μm, 4 μm, 6 μm, 8 μm, 10 μm. The surface morphology is shown in Figure 7a1–a5. As is shown in Figure 7a1–a5, with the decrease of the scanning interval, the ridges, and inhomogeneity brought by the scanning interval gradually disappear. Combined with Figure 8a, the roughness also begins to decrease gradually. However, as the scanning interval becomes smaller, the speed of roughness reduction also begins to decrease. This is because when the scanning interval decreases to a certain extent, the effect of the scanning interval on the surface roughness begins to decline. Further reducing the scanning interval has no obvious effect on improving the surface quality but seriously affects the polishing efficiency. Although the surface quality is slightly improved when the scanning interval is 2 μm, the scanning time is doubled. Therefore, by weighing the polishing efficiency and polishing effect, we selected 4 μm as the optimal scanning interval.

The influence of laser fluence on the surface morphology and the surface roughness is investigated in Figure 7b1–b4 and Figure 8b. The RB-SiC surface is irradiated by a femtosecond laser with a repetition frequency of 100 kHz, a scanning velocity of 150 mm/s, scanning times of 2, a scanning interval of 4 μm, and different laser fluences of 0.52 J/cm^2^, 0.57 J/cm^2^, 0.62 J/cm^2^, 0.67 J/cm^2^, and 0.72 J/cm^2^, respectively. As shown in Figure 7b1–b5 and Figure 8b, with the increase of laser fluence, the ridges on the surface gradually disappear and the surface morphology becomes uniform, but the surface roughness decreases first and then increases, reaching its lowest value when the laser fluence is 0.62 J/cm^2^. When scanning with a laser fluence slightly above the ablation threshold, some defects and pits were removed and the surface quality improved. With the increase in laser fluence, the defects and pits have completely disappeared, and the ridges formed during the scanning process have become the main factor affecting the surface quality. When the laser fluence increased to 0.62 J/cm^2^, even though there were still ridges formed by scanning, the surface roughness reached its minimum. When the laser fluence continues to increase due to the high laser fluence, the material removed gradually increases, and phases of explosion, ablation, vaporization, and re-solidification will occur on the surface, causing the filling of defects and pits with vaporized and re-solidified powder. Materials that solidify after vaporization will seriously affect the surface quality after polishing, resulting in a rapid increase in surface roughness.

Therefore, considering the surface morphology and surface roughness after polishing, the best scanning interval and laser fluence were selected as 4 μm and 0.62 J/cm^2^.

### 3.3. Influence of Equivalent Pulse Numbers on Polishing

**Figure 9:**
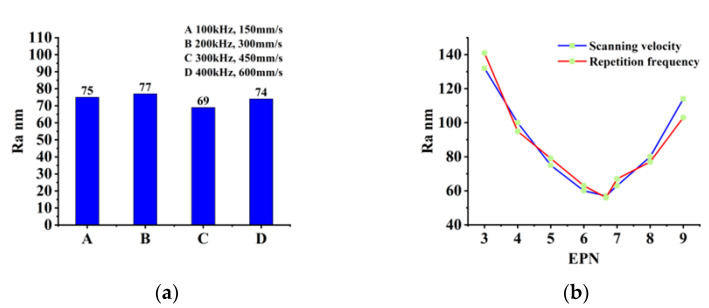
Curve of surface roughness with the change of different polishing parameters: (**a**) Same EPN with different repetition frequency and scanning velocity; (**b**) Different EPN.

According to Formula (7), the EPN is determined by repetition frequency, scanning velocity, and beam diameter. Generally, the beam diameter is controlled by the defocus. In this experiment, the F-theta mirror can only ensure a uniform beam diameter in the focal plane, so we fixed the beam diameter at 10 μm, which is the beam diameter in the focal plane. Figure 9a shows the surface roughness values obtained at several different repetition frequencies and scanning velocities with an EPN of 6.67, a scanning interval of 4 μm, a scanning time of 2, and a laser fluence of 0.62 J/cm^2^. There is little roughness difference between the groups of experiments. When the EPN does not change, the variation in scanning velocity and repetition frequency has no effect on surface roughness.

### 3.4. Influence of Scanning Model and Times on Polishing

**Figure 10:**
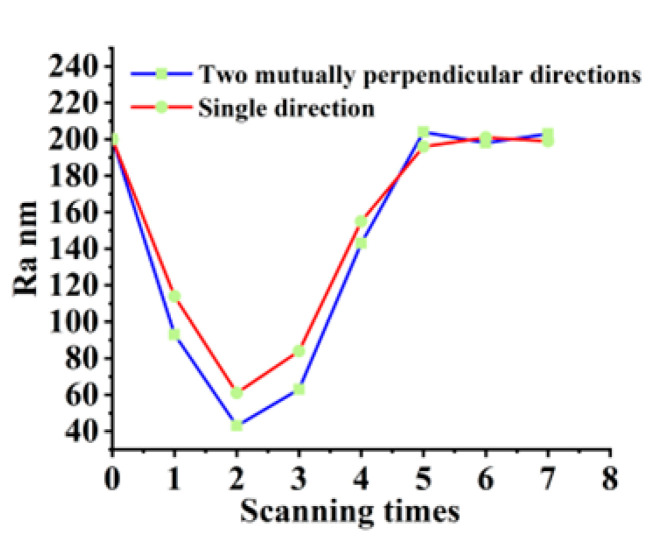
Curve of surface roughness with the change of scanning times in different model.

In the following experiments, laser fluence of 0.62 J/cm^2^, scanning interval of 4 μm, scanning velocity of 150 mm/s, and repetition frequency of 100 kHz are selected as the best polishing parameters. The influence of the scanning model and times on the surface morphology and roughness is investigated in Figure 10. However, the surface quality obtained by scanning two directions perpendicular to each other is better. When measuring the roughness, we need to measure the roughness in different directions of the polished surface, find its mean value, and take it as the final surface roughness. When the laser scans in only one direction, the average surface roughness obtained is higher because the ridges generated during the scanning process will affect the roughness perpendicular to the scanning direction. Therefore, we can obtain a lower surface roughness by scanning along two mutually perpendicular directions.

As can be seen from Figure 10, when other parameters are optimal polishing parameters and remain unchanged, the surface roughness decreases first and then increases with the increase in scanning times. With the increase in the number of scans, the surface bumps and defects are gradually removed. Continued scanning causes the powder created by the removed material to recondense [34], at which point the femtosecond laser exerts its welding action.

## 4. Discussion

**Figure 11:**
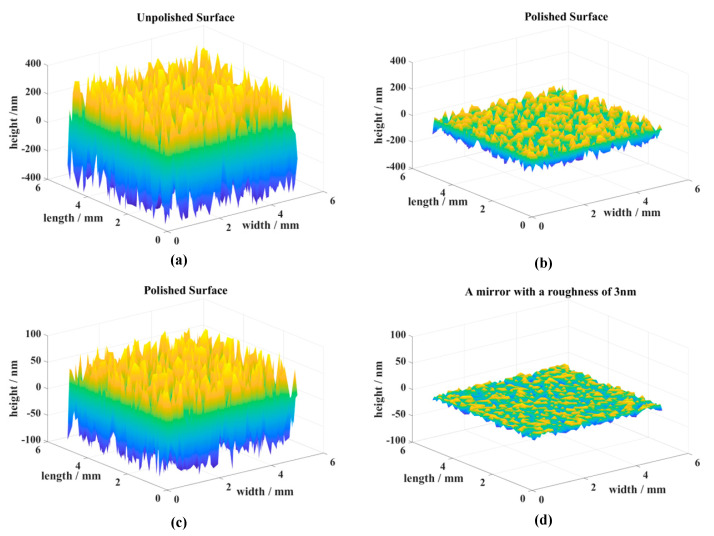
Surface morphology of initial surface, polished surface, and mirror surface: (**a**) Initial surface; (**b**,**c**) Polished surface; (**d**) Mirror surface.

According to the above analysis, after optimizing the parameters, femtosecond laser polishing can effectively reduce the surface roughness of RB-SiC. A laser fluence of 0.62 J/cm^2^, a scanning interval of 4 μm, scanning times of 2, a scanning velocity of 150 mm/s, and a repetition frequency of 100 KHz can be selected as the optimal scanning parameters. After scanning two times along two mutually perpendicular directions with the above polishing parameters, a surface with a roughness value Ra of 43 nm can be obtained. Figure 11 shows the surface morphology of the different surfaces simulated by point-by-point measurements. Figure 11a,b show the surface morphology before and after polishing, respectively. There is a significant reduction in surface roughness, and the ridges formed by scanning are almost unobservable after optimization of the polishing parameters. Figure 11b,c show the same surface at different scales. In order to compare the surface polished by a femtosecond laser with the RB-SiC mirror in practice, we also measured the RB-SiC planar mirror after optical coating. By comparison with Figure 11c,d, there is an obvious gap in surface morphology and roughness between the surface polished by a femtosecond laser and the plane mirror in a practical application.

Table 3 shows the polishing process of diamond abrasive particles for an RB-SiC disc with a diameter of 60 mm, which takes 9 h for the surface roughness to reach 41 nm [35]. The polishing parameters were set to the optimal polishing parameters, the surface roughness reached 43 nm by femtosecond laser polishing, and the polishing time was 2.6 h for a disc with a diameter of 60 mm. Under the same initial conditions and the same surface roughness after polishing, the time used for femtosecond laser polishing is about 28% of that for abrasive polishing, and the polishing efficiency is greatly improved.

Thus, laser polishing of RB-SiC is expected to replace some of the steps in mechanical polishing, which can greatly improve the polishing efficiency.

## 5. Conclusions

In this paper, we focus on the optimization of parameters during the polishing of a RB-SiC surface with a femtosecond laser. The type of ablation in the femtosecond laser polishing process is analyzed theoretically, and the single-pulse ablation threshold of SiC is calculated at 0.41 J/cm^2^. In addition, the effects of polishing parameters on the surface roughness after polishing were analyzed through a series of experiments. When the laser fluence of 0.62 J/cm^2^, the scanning velocity of 150 mm/s, the repetition frequency of 100 kHz, and the scanning interval of 4 µm were selected as the polishing parameters, the surface roughness Ra was reduced from 200 ± 20 nm to 43 nm after scanning twice along two mutually perpendicular directions.

The experimental results show that the femtosecond laser polishing proposed in this paper significantly improves the surface quality and polishing efficiency, and it is expected to replace part of the traditional mechanical polishing process. Femtosecond laser polishing has the advantages of simplicity, flexibility, and low requirements for experimental environments. It provides a new idea for high-performance mirror polishing of RB-SiC, which is beneficial to the development of ultra-precision processing.

There are many shortcomings in this experiment. Due to the limitations of the experimental conditions, we were unable to analyze the chemical composition of the polished surface, which severely restricted us from performing higher precision polishing. Restricted by the scanning equipment, we were unable to fully utilize the energy of the laser by defocusing, which limited the further improvement of the scanning efficiency. In future research, we should establish the corresponding simulation model and experimental conditions to analyze the nonlinear effects of wavelength and pulse duration on the polishing effect by deeply investigating the femtosecond laser ablation mechanism. Further, optimizing the experimental design to improve the polishing efficiency while considering the polishing effect is also the focus of future research.

## Figures and Tables

**Table 1:** Femtosecond parameters for polishing processing.

Parameters	Value
Wavelength λ	515 nm
Beam polarization	Linear
Pulse energy	0~100 μJ
The full width at half maximum (FWHM) pulse duration $t_{P}$	282 fs
Laser beam diameter $\omega_{0}\left(1/e^{2}\right)$	10 μm
Frequency f	60~1000 kHz
Maximum scanning velocity	7000 mm/s
Focal length of the F-theta-lens $f_{1}$	100 mm

**Table 2:** Thermophysical parameters of SiC.

Parameters	Symbol	SiC [31]
Electron-phonon coupling strength $\left[W/(m^{3}\cdot K)\right]$	g	1.12 × 10^17^
Heat capacity of electron $\left[J/(m^{3}\cdot K)\right]$	$C_{e}$	78.3
Thermal conductivity of electron [J/(m⋅K⋅s)]	$K_{e}$	27.3
Heat capacity of lattice $\left[J/(m^{3}\cdot K)\right]$	$C_{l}$	1.659 × 10^5^
Thermal conductivity of lattice [J/(m⋅K⋅s)]	$K_{l}$	324
Reflectivity [1]	R	0.8
Thermodynamic equilibrium critical temperature [K]	$T_{t}$	3973
Depth of scanning trajectory $L_{p}$	$L_{p}$	1.37 × 10^−9^

**Table 3:** Abrasive polishing process [35].

Step	Abrasive Grain Size	Surface Roughness (Rq)	Time
1	W20	162.2 nm	0.5 h
2	W7	162.2–56.1 nm	0.5 h
3	W7	56.1–41.306 nm	8 h
4	W0.5	41.036–18.753 nm	4 h
5	W0.5	18.753–1.525 nm	10 h

## Data Availability

Not applicable.

## References

[1] Redding D., Feinberg L., Postman M., Stahl H.P., Stahle C., Thronson H. (2014). Beyond JWST: Performance Requirements for a Future Large UVOIR Space Telescope.

[2] Nguyen T., Liu D., Thongkaew K., Li H., Qi H., Wang J. (2018). The wear mechanisms of reaction bonded silicon carbide under abrasive polishing and slurry jet impact conditions. Wear.

[3] Rajak D.K., Wagh P.H., Linul E. (2022). A review on synthetic fibers for polymer matrix composites: Performance, failure modes and applications. Materials.

[4] Brinksmeier E., Riemer O., Gessenharter A. (2006). Finishing of structured surfaces by abrasive polishing. Precis. Eng..

[5] Hashmi A.W., Mali H.S., Meena A., Saxena K.K., Puerta A.P.V., Prakash C., Buddhi D., Davim J., Abdul-Zahra D.S. (2022). Understanding the mechanism of abrasive-based finishing processes using mathematical modeling and numerical simulation. Metals.

[6] Vora H., Orent T.W., Stokes R.I. (1982). Mechanochemical Polishing of Silicon Nitride. J. Am. Ceram. Soc..

[7] Nowak K.M., Baker H.J., Hall D.R. (2006). Efficient laser polishing of silica micro-optic components. Applied Optics.

[8] Mi S., Toros A., Graziosi T., Quack N. (2019). Non-contact polishing of single crystal diamond by ion beam etching. Diam. Relat. Mater..

[9] Cheng H., Feng Z., Wang Y., Lei S. (2005). Magnetorheological Finishing of SiC Aspheric Mirrors. Mater. Manuf. Process..

[10] Yuan Z., He Y., Sun X., Wen Q.J.M. (2018). UV-TiO2 photocatalysis-assisted chemical mechanical polishing 4H-SiC wafer. Mater. Manuf. Process..

[11] Kubota A., Nagae S., Motoyama S. (2020). High-precision mechanical polishing method for diamond substrate using micron-sized diamond abrasive grains. Diam. Relat. Mater..

[12] Tam H.Y., Cheng H.B., Wang Y.W. (2007). Removal rate and surface roughness in the lapping and polishing of RB-SiC optical components. J. Mater. Process. Technol..

[13] Zhao J., Zhan J., Jin R., Tao M. (2000). An oblique ultrasonic polishing method by robot for free-form surfaces. Int. J. Mach. Tools Manuf..

[14] Temmler A., Willenborg E., Wissenbach K. (2012). Laser Polishing.

[15] Shao Y., Sun S.-F., Liu G.-L., Wang P.-P., Shao J., Zhang F.-Y., Wang X. (2021). Laser-assisted thermochemical ultrahigh-precision polishing of titanium in phosphoric acid solution. Int. J. Adv. Manuf. Technol..

[16] Weingarten C., Heidrich S., Wu Y., Willenborg E. (2015). Laser Polishing of Glass.

[17] Sassmannshausen A., Brenner A., Finger J. (2021). Ultrashort pulse laser polishing by continuous surface melting. J. Mater. Process. Technol..

[18] Temmler A., Liu D., Luo J., Poprawe R. (2020). Influence of pulse duration and pulse frequency on micro-roughness for laser micro polishing (LµP) of stainless steel AISI 410. Appl. Surf. Sci..

[19] Zhang R., Huang C., Wang J., Chu D., Liu D., Feng S. (2022). Experimental investigation and optimization of femtosecond laser processing parameters of silicon carbide–based on response surface methodology. Ceram. Int..

[20] Qu Y., Choi H., Perry T., Jeon Y., Pfefferkorn F., Li X., Duffie N. Numerical and Experimental Investigation of Micromelting for Laser Micro Polishing of Meso/Micro Metallic Components. Proceedings of the ASME 2006 International Manufacturing Science and Engineering Conference.

[21] Chen H., Wei C., Cao Z., Peng X., Jiang Z., Shao J.J.O.M.E. (2022). Femtosecond laser-selective polishing of RB-SiC at a fluence between its two-phase threshold. Opt. Mater. Express.

[22] Gamaly E.G., Luther-Davies B., Kolev V.Z., Madsen N.R., Duering M., Rode A.V. (2005). Ablation of metals with picosecond laser pulses: Evidence of long-lived non-equilibrium surface states. Laser Part. Beams.

[23] Bulgakova N.M., Stoian R., Rosenfeld A., Hertel I.V., Marine W., Campbell E.E.B. (2005). A general continuum approach to describe fast electronic transport in pulsed laser irradiated materials: The problem of Coulomb explosion. Appl. Phys. A.

[24] Anisimov S., Kapeliovich B., Perelman T. (1974). Electron emission from metal surfaces exposed to ultrashort laser pulses. Zh. Eksp. Teor. Fiz..

[25] Christensen B.H., Vestentoft K., Balling P. (2007). Short-pulse ablation rates and the two-temperature model. Appl. Surf. Sci..

[26] Bussière B., Sanner N., Sentis M., Utéza O. (2017). Importance of surface topography on pulsed laser-induced damage threshold of Sapphire crystals. Sci. Rep..

[27] Zheng Q., Fan Z., Jiang G., Pan A., Yan Z., Lin Q., Cui J., Wang W., Mei X. (2019). Mechanism and morphology control of underwater femtosecond laser microgrooving of silicon carbide ceramics. Opt. Express.

[28] Mizoshiri M., Yoshidomi K. (2021). Cu Patterning Using Femtosecond Laser Reductive Sintering of CuO Nanoparticles under Inert Gas Injection. Materials.

[29] Richerzhagen B., Kutsuna M., Okada H., Ikeda T. (2003). Water-Jet-Guided Laser Processing.

[30] Peng A., Peng Y., Zhang Y., Li K., Chen J. (2006). Absorption coefficient and damage threshold in single-crystalline silicon with femtosecond laser. J. Henan Norm. Univ. (Nat. Sci.).

[31] Zhang H.Z., Wang H.Y., Liu F.F., Wang L. (2020). Investigation on femtosecond laser ablative processing of SiCp/AA2024 composites. J. Manuf. Process..

[32] Kelly R., Miotello A. (1996). Comments on explosive mechanisms of laser sputtering. Appl. Surf. Sci..

[33] Dong Y., Molian P. (2003). Femtosecond pulsed laser ablation of 3CSiC thin film on silicon. Appl. Phys. A.

[34] Luo J., Zhou K., Ma Y., Lei Y., Liu H., Tong H., Xiao R., Wang Y., Chen Y., Chen Z. (2022). Femtosecond laser welding for robust and low loss optical fiber bonding. Opt. Express.

[35] Kang N., Li S., Zheng Z., Dai Y. (2008). Experimental study on super smooth polishing of typical SIC optical materials. China Mech. Eng..

